# Enlisting the support of trusted sources to tackle policy problems: The case of antimicrobial resistance

**DOI:** 10.1371/journal.pone.0212993

**Published:** 2019-03-21

**Authors:** Aaron Martin, Timothy B. Gravelle, Erik Baekkeskov, Jenny Lewis, Yoshi Kashima

**Affiliations:** 1 School of Social and Political Sciences, Faculty of Arts, University of Melbourne, Victoria, Australia; 2 Institute for Social Science Research, University of Queensland, Queensland, Australia; 3 Melbourne School of Psychological Sciences, Faculty of Medicine, Dentistry and Health Sciences, University of Melbourne, Victoria, Australia; University of Turin, ITALY

## Abstract

Antimicrobial resistance represents one of the world’s most pressing public health problems. Governments around the world have—and will continue to—develop policy proposals to deal with this problem. However, the capacity of government will be constrained by very low levels of trust in government. This stands in contrast to ‘medical scientists’ who are highly trusted by the public. This article tests to what extent trusted sources can alter attitudes towards a policy proposal to regulate the use of antibiotics. We find that respondents are much more likely to support a policy put forward by ‘medical scientists.’ This article provides some initial evidence that medical scientists could be used to gain support for policies to tackle pressing policy challenges such as AMR.

## Introduction

Antimicrobial resistance (AMR) is now recognized as one of the world’s most pressing public health problems [1,2]. Governments around the world have—and will continue to—develop policy proposals to deal with this problem. However, the capacity of government will be constrained by levels of trust in government. Trust in government has been declining around the world in recent years [3–6]. Kettl [7] argues that ‘the rising tide of distrust in government is surely one of the biggest challenges facing the world’s democracies in the twenty-first century.’ In such an environment, government may find it harder to secure support for policy proposals to deal with AMR. At a time when trust in government is declining and social problems (such as AMR) are becoming more complex, it is likely that government will have to enlist the support of trusted sources to secure support for policy reforms. This article tests to what extent trusted sources can alter attitudes toward a policy proposal to regulate the use of antibiotics.

## Trusted sources and AMR

### Trust in government and scientists

While it is clear that trust in government in many countries is at historically low levels [7,8], a less remarked-upon pattern is that not all authorities are distrusted to the extent that government is. This is especially true when we compare the authorities manipulated in this experiment: government versus scientists (as explained in the methods section below). Public opinion data from the United States shows that ‘medical scientists’ are ranked as the *most* trustworthy of all professions [9]. These findings have been replicated cross-nationally [10]. Taken together this research provides very strong evidence that while scientists are highly trusted, government is not.

The differences between trust in government and trust in scientists could have important consequences. Source effects have been of long-standing interest across the social sciences [11]. Sources have been found to have the largest effect on attitudes when they are considered credible and trustworthy [12–14]. In policy contexts we would expect little-trusted politicians to be a less effective source for policy messaging than other sources, such as ‘scientists’. Given that trust varies by source, the way source shapes support for policy is an important question. Therefore, we expect that citizens will be more likely to support a policy proposal put forward by a trusted source. Because source effects are complex [15] it is important to investigate this experimentally for the particular case of AMR.

We test this hypothesis using a unique survey experiment fielded in Australia. While we limit our study to one country, we believe this case is instructive for a number of reasons. First, Australia has experienced the same steep declines in political trust experienced in other western democracies [6]. Second, we also find the same patterns in Australia in terms of scientists being seen as trustworthy and contributing positively to society–much more so than government [16,17]. Australia therefore provides a useful test case for the broader phenomenon where trust in government is low (and declining), but scientists are highly trusted.

### Antimicrobial resistance

Antibiotic and other antimicrobial resistance (AMR) has been known since antibiotics were discovered in the 1940s [18] Yet it is now a rapidly emerging area for public policy-making, in Australia and globally [1,19,2]. Species of bacteria and other microbes evolve under the selection pressures imposed by antibiotics and other antimicrobials to become resistant to such interventions. In turn, evolution renders these mainstays of human and veterinary medicine less effective. AMR also has implications for food safety. As animals and crops raised in close proximity become less protectable from the spread of microbials, contemporary intensive mass production of foodstuffs becomes less viable. The Australian government is one of many across the globe recognising that ‘the appropriate and judicious use of antimicrobials is essential to slowing the emergence of resistance’ of microbes to antimicrobials [20,1]. In particular, limiting uses of antibiotics to the most necessary medical applications is one of the key public policies for curbing AMR.

In our experiment, we measure support for a policy proposal to regulate and monitor the use of antibiotics. AMR is an interesting policy area because we would expect *ex-ante* that citizens would be supportive of any plausible attempt to curb it. In this sense the attitudes towards curbing AMR may be regarded as a ‘hard-test’ of the expectation that the public will support a policy reform put forward by scientists. It is not a politically contested issue like immigration or climate change [21–24], nor very controversial.

AMR has received repeated attention from scholars at the intersection of social science and medicine in the last two decades [25–27]. Prior policy-related research has looked at factors diverse as pharmacists’ administering of antibiotics [28], antibiotics options markets [27], and antibiotic use in low income countries [25]. Even more specific to our focus are surveys and more recently citizen juries that have been used to identify drivers of public demand [29–31] and of physicians’ prescribing patterns [32–33]. Experimental and observational studies of public information impact on uses of antibiotics have identified that information campaigns *can* limit popular demand for or physicians’ propensity to prescribe antibiotics [34,35]; yet there is little clarity about ‘which [information campaign] strategy is most efficient in changing attitudes and practices’ [36].

### Examining the effect of trusted sources

No research (that we are aware of) has been done to test the effect of enlisting trusted sources to gain support for a policy to curb AMR. We hypothesise that one consequence of declining support for government may be that citizens are more likely to support a policy promoted by a ‘non-political actor,’ rather than government. For AMR in particular, we expect a policy proposal put forward by ‘medical scientists’ will garner more support than a policy proposal put forward by ‘government.’ Beyond this, we also test how policy support is shaped by the organisation’s (whether ‘medical scientists’ or ‘government’) perceived competence in delivering effective policy. Literature has also shown that competence can have an effect on political trust [5] which may in turn have an effect on support for policy. We examine this by sending signals about the level of competence of the two different sources.

Based on this literature we tested two hypotheses:

H1: A policy proposal from a trusted source (medical scientists) will receive higher support than a proposal from a less trusted source (the government in Canberra).
H2: A policy proposal coming from a source that is signalled to be competent will receive higher support.

## Data and methods

To test these theoretical expectations, we designed an online survey experiment, which we administered to a demographically representative non-probability sample of the Australian public (n = 388) through Qualtrics, an online survey platform. The sample (derived from an online panel of respondents supplied by Qualtrics) was balanced on age, gender, educational attainment, and party identification (the latter was matched to 2016 Australian Election Study data). This study was approved by the Human Ethics Committee at the University of Melbourne, Australia.

The use online non-probability samples in quantitative social science has expanded substantially in the past decade. While some studies find that probability and non-probability samples yield different estimates [37,38], there is growing evidence that there are few meaningful differences between the inferences obtained from online non-probability panels and probability-based samples [39–41]. Our experimental design also means that random assignment is far more important than random selection. Our sample is thus fit for purpose [42].

The experiment was designed as a 2 x 2 factorial experiment involving two treatments: (1) a federal government/medical scientist framing of the policy proposal, and (2) a competence frame highlighting the policy expertise and effectiveness of the federal government/medical scientists (versus a control condition where nothing about competence is presented to the respondent). Full details of the experiment (including the full survey questionnaire) are provided in the appendix.

After a generic description of the dangers and causes of anti-microbial resistance, respondents were presented with a policy proposal from either ‘the government in Canberra’ or ‘Australia’s leading medical scientists.’ Specifically, respondents were told that:

**[The government in Canberra/Australia’s leading medical scientists]** believe that an effective way to address this problem is to more closely monitor and regulate how medical professionals prescribe antibiotics. Accordingly, **[the government in Canberra/Australia’s leading medical scientists]** have put forward a proposal whereby they would monitor how many antibiotics medical professionals are prescribing and, where necessary, enforce that they prescribe less.

Respondents assigned to receive the competence treatment were further told that:

**[The government in Canberra/Australia’s leading medical scientists]** has a long and strong record of successfully delivering programs like this. For example, in 2016 they successfully monitored a program which asked surgeons to fill out a checklist after every procedure to ensure no mistakes were made. This was estimated to have saved 23,000 lives and prevented over 40,000 infections. This track record shows that the program is likely to be a success.

We then presented respondents with a series of comprehension checks to confirm that respondents had read the article properly–based on which we deleted 12 observations from those who failed to correctly identify what topic (i.e. AMR) the article referred to; all respondents correctly answered the second manipulation check asking who the actor advocating for the policy (i.e. government versus medical scientists) was. Respondents were then asked about our key outcome measure: ‘Do you support the proposal for [the government in Canberra/Australia’s leading medical scientists] to monitor how many antibiotics medical professionals are prescribing and, where necessary, enforce that they prescribe less?’ Response categories were a four-point scale from ‘Strongly agree’ to ‘Strongly disagree.’

For our statistical analysis, we first examine the distribution of responses across the two experimental conditions in simple bivariate tables. Given the four ordered categories of the dependent variable, we then model the effects of the trusted source and competence frames using ordinal logistic regression [43]. The ordinal logit coefficients are thus our estimates of the effects of the two experimentally-manipulated frames on the cumulative log odds of providing a higher response on the dependent variable (i.e., expressing greater support for the proposal), controlling for party identification. For ease of interpretation, the effects of the two experimental frames obtained from the ordinal logistic regression results are also reported as predicted probabilities [44].

## Results

Examining first the bivariate distributions for support for prescription monitoring by each treatment, it is clear that the proposal enjoys majority support regardless of how it is framed. Overall support (for the entire sample) is 91.8 percent (see Table 1). Still, the framing of the proposal does strengthen or weaken the level of support. Framing the proposal as coming from the government reduces aggregate support to 85.1 percent (versus 98.5 percent when the proposal is framed as coming from Australia’s leading medical scientists). The modal response also shifts from supporting the proposal ‘strongly’ when presented as originating with Australia’s leading medical scientists to weaker (‘somewhat’) support when presented as a government proposal. By contrast, the competence frame increases support to 94.8 percent (versus 88.7 percent when no competence frame is presented). The modal response similarly shifts from ‘somewhat’ to ‘strongly’ supporting the proposal with the competence frame.

**Table 1:** Support for monitoring the use of antibiotic monitoring.

	Government Frame (%)	Medical Scientist Frame (%)	Competence Frame (%)	No Competence Frame (%)	Total (%)
Strongly support	30.4	59.8	53.9	36.4	45.1
Somewhat support	54.6	38.7	40.9	52.3	46.7
Somewhat oppose	12.4	1.6	3.6	3.6	7.0
Strongly oppose	2.6	0.0	1.6	1.0	1.3
n	194	194	195	193	388
χ^2^ (3)	45.21***		15.59**		

** *p* ≤ 0.01

*** *p* ≤ 0.001

It is notable that while the government frame reduces support for antibiotic monitoring, highlighting organisational competence appears to (at least partly) counteract this negative effect. To reconfirm this result, and to understand how the two frames work in combination with each other (and with other factors, such as party identification), multivariate analysis is required. The results from the ordinal logit models indicate that both the government frame and competence frame continue to exert statistically significant effects when evaluated in tandem (see Table 2). The government frame (contrasted to the medical scientist frame) reduces the cumulative log odds of supporting the antibiotic monitoring proposal by 1.42 (95% c.i.: -1.84 to -0.99). As suggested above, this negative effect is partly (though not completely) mitigated by the competence frame, which increases the cumulative log odds of support by 0.84 (95% c.i.: 0.43 to 1.25). Incidentally, additional analyses not reported here fail to find a significant interaction between the government and competence frames–that is, there is no evidence that the competence frame exerts a different effect when combined with the government frame or the medical scientist frame. While we may expect those who identify with the Liberal Party (the current party in power) to have greater support for the policy proposal we find that party identification has no effect on the responses.

**Table 2:** Explaining support for antibiotic monitoring (ordinal logit).

	b	(SE)	95% c.i.		
Intercept 4	0.15	0.25	-0.33	0.63	
Intercept 3	3.06	0.30	2.46	3.66	***
Intercept 2	5.04	0.51	4.04	6.05	***
Government in Canberra frame	-1.42	0.21	-1.84	-0.99	***
Competence frame	0.84	0.21	0.43	1.25	***
Party Identification (ref = Labor)					
Liberal-National	0.00	0.25	-0.49	0.49	
Other party/No party	-0.36	0.27	-0.89	0.17	

* *p* ≤ 0.05

** *p* ≤ 0.01

*** *p* ≤ 0.001

The results of the regression analysis are easier to interpret when translated into predicted probabilities on a 0–1 scale (see Table 3). Presented in this way, the results reconfirm those from Table 1 and Table 2. Specifically, framing the proposal as coming from the government (as opposed to medical scientists) reduces the predicted probability of strongly supporting the proposal by 0.34. By contrast, the competence frame raises the predicted probability of strongly supporting the proposal by 0.20. In short, drawing attention to the competence of the source supporting the antibiotic monitoring proposal does not fully make up for the loss of support when it comes from the government.

**Table 3:** Predicted probabilities of support for antibiotic monitoring.

	Government Frame	Medical Scientist Frame	Change		Competence Frame	No Competence Frame	Change	
p(Strongly Support)	0.28	0.61	-0.34	***	0.54	0.34	0.20	***
p(Somewhat Support	0.60	0.35	0.25	***	0.41	0.56	-0.15	***
p(Somewhat Oppose)	0.10	0.03	0.08	***	0.04	0.08	-0.04	***
p(Strongly Oppose)	0.02	0.00	0.01	*	0.01	0.01	-0.01	*

* *p* ≤ 0.05

*** *p* ≤ 0.001

## Discussion

Political trust is important because it has been shown to have numerous attitudinal and behavioural consequences [4,5,45]. In this article we have explored one such consequence: whether an AMR policy proposal put forward by scientists is more likely to be supported than one put forward by government. As we noted above, the experiment was in a lot of ways a ‘hard-test’ given that most people might recognise AMR as a pressing public health problem and be supportive of any attempt to curb it. This is borne out, with high aggregate levels of support for the policy. Even allowing for that, we find a significantly higher level of support for the policy proposal put forward by scientists, as opposed to government.

What mechanisms may be driving this result? While we have not been able to test this in the current study, it seems likely that the term ‘medial scientists’ triggers a number of positive associations in the mind of the public, whereas ‘government’ triggers a number of negative associations. Medical scientists also hold a special status in health policy—medical expertise is important, strongly legitimised through institutions, and embedded throughout policy networks [46]. One might expect the medical scientists to be more trusted by the public because of this. We have some additional evidence that trust is in fact the mediating factor with trust in the effectiveness of the actor in administering this policy (which is a partial proxy for general trust in the actor) being much higher in the medical scientist condition as opposed to the government condition. (In our survey asked the following post-treatment question: How much confidence do you have in the [federal government in Canberra/Australia’s leading medical scientists] to EFFECTIVELY monitor and enforce the use of antibiotics?’ While 49 percent of respondents receiving the ‘Australia’s leading medical scientists’ condition expressed ‘a lot of trust’, only 13 percent of respondents receiving the ‘federal government in Canberra’ condition responded in the same way (χ^2^ = 79.59, *d*.*f*. = 3; *p* < 0.001). This, of course, is not general trust and is conditioned on treatment. Still, this provides additional evidence of the potential causal mechanism). The results support our hypothesis (and the literature on source effects) that policy proposals put forward by trusted sources are much more likely to be supported. Future research ought to test trustworthiness of particular actors as a mediating variable.

Larger questions remain that we think are fruitful avenues for future research. For example, while we expect the findings here to be replicated in other settings where government is distrusted and scientists are trusted, this remains a hypothesis until we have data to confirm this. Coordination of a policy regulating antibiotic use is also complicated by the number of individuals involved in administering antibiotics—Broom, Broom Kirby and Scambler [26] show that antibiotic use is an interprofessional rather than a purely medical problem. There are also broader normative questions about whether it is desirable for medical scientists to be engaged in such policy advocacy and implementation.

There are of course several limitations to this study. This study employs a medium-sized sample in a particular setting. The effects we find may vary across issue and country. For example, we don’t know what happens when respondents are confronted with more controversial policies concerning issues such as immigration or climate change where respondents may have strong priors or take cues from party leaders. We have also not tested what happens to the reputation of scientists if and when they enter political debate. That said, for an uncontroversial issue like AMR there may not be much reputational damage.

Even allowing for these limitations this article has provided credible evidence that medical scientists could be used to gain support for policies that will be essential if AMR is to be curbed. This article shows that the public are much more likely to support a policy proposal to deal with AMR put forward by ‘scientists’ as opposed to ‘government.’ It also shows that drawing attention to the competence of medical scientists or government alters attitudes towards the policy proposal. In an environment of growing distrust in government alongside pressing public health problems such as AMR it is essential that such questions are explored.

## Supporting information

S1 Dataset PLOS_ONE_20180131. (CSV)

S2 Dataset PLOS_ONE_20180131. (DTA)
